# Continuous versus intermittent beta-lactam antibiotic infusions in critically ill patients: The UK cohort of the BLING III trial

**DOI:** 10.1177/17511437251396871

**Published:** 2025-11-27

**Authors:** Janis Best-Lane, Farah Al-Beidh, Greg Barton, Dorrilyn Rajbhandari, Xiaoqiu Liu, Jayanthi Mysore, Serena Knowles, Naomi Hammond, Joel Dulhunty, John Myburgh, Jeffrey Lipman, Stephen J. Brett

**Affiliations:** 1 Imperial Clinical Trials Unit, Imperial College London, UK; 2 Mersey and West Lancashire Teaching Hospitals NHS Trust, Prescot, UK; 3 Critical Care Program, The George Institute for Global Health, UNSW Sydney, Australia; 4 Malcolm Fisher Department of Intensive Care, Royal North Shore Hospital, St Leonards, NSW, Australia; 5 University of Queensland, Brisbane, Australia; 6 Royal Brisbane and Women’s Hospital, QLD, Australia; 7 Redcliffe Hospital, Brisbane, QLD, Australia; 8 St George Hospital, Sydney, NSW, Australia; 9 Jamieson Trauma Institute, Brisbane, QLD, Australia; 10 Division of Anesthesia, Critical Care, Emergency and Pain Medicine, University of Montpellier, Nîmes University Hospital, France; 11 Division of Anaesthetics, Pain Medicine and Intensive Care, Department of Surgery and Cancer, Imperial College London, UK; 12 Imperial College Healthcare NHS Trust, London, UK

**Keywords:** antibiotic, clinical outcomes, infection, intensive care, microbiology, pharmacokinetics, process of care

## Abstract

**Background:**

There are theoretical reasons why beta-lactam antibiotics may be more effective in treating severe infections if administered by continuous infusion, rather than short intermittent infusions. The Beta-Lactam Infusion Group (BLING) III trial was a multinational randomised clinical trial (RCT) which tested this hypothesis in participants with sepsis who were cared for in an intensive care unit (ICU). The United Kingdom (UK) findings are reported here.

**Methods:**

The global trial was an open-label RCT conducted in the UK, Australia, New Zealand, Belgium, France, Sweden and Malaysia. Participants were critically ill adults being treated with meropenem or piperacillin/tazobactam due to a confirmed or presumed infection. Participants were randomised to receive the antibiotic by either continuous infusion or short intermittent infusion at equivalent daily doses as selected by the treating team. The primary outcome was 90-day all-cause mortality; secondary outcomes included clinical cure up to 14 days after randomisation, new infection and acquisition of resistant organisms, ICU and in hospital mortality.

**Results:**

Overall, 7202 participants were randomised, with 2900 from the UK. The UK cohort had very similar baseline characteristics and outcomes to the global trial. For continuous versus intermittent infusion, the global trial showed 24.9% versus 26.8% participants had died by 90 days (odds ratio 0.91, 95% CI: 0.81–1.01, *p* = 0.08); and in the UK 26.7% versus 29.3% participants had died (odds ratio 0.88, 95% CI: 0.75–1.04, *p* = 0.13). Although not statistically significant, all outcomes showed point estimates in favour of continuous infusion.

**Conclusions:**

The findings in the UK cohort are consistent with the conclusions drawn from the global BLING III trial. It seems reasonable to conclude the finding are applicable to the UK.

## Introduction

The Beta-Lactam Infusion Group (BLING) III trial was a global clinical trial of continuous versus intermittent infusions of beta-lactam antibiotics in critically ill patients with sepsis.^1^ The trial included 7202 randomised participants of whom a significant proportion were from the United Kingdom (UK; 40.3%). This article, and supplemental material, presents the data from the UK that formed part of the global dataset.

Beta-lactam antibiotics such as meropenem and piperacillin-tazobactam are commonly used to treat severe infections in intensive care. These antibiotics have ‘time-dependent’ kill-characteristics such that are they better at killing bacteria if the concentration of drug at the target site is optimised for prolonged periods of time. Recent data have confirmed that continuous infusions of beta-lactam antibiotics resulted in levels above the minimum inhibitory concentration of typical pathogens.^2^ Thus, there are sound theoretical reasons why continuous infusions of beta-lactam antibiotics should be clinically more effective than intermittent injections or short duration intermittent infusions. Prior studies had been somewhat supportive of this premise, but conclusive evidence was lacking.^2–4^

Thus, the BLING III trial was conducted by an international group to test the hypothesis that continuous infusion of meropenem and piperacillin-tazobactam resulted in decreased all-cause mortality at 90 days in critically ill patients with sepsis compared with short intermittent infusion. Key secondary outcomes were test of cure at 14 days post randomisation, acquisition of resistant organisms, and lengths of intensive care unit (ICU) and hospital stay. Tertiary outcomes included days alive and free of ICU, hospital, ventilatory and renal support.

Overall, the BLING III trial did not show a statistical difference in primary outcome in the unadjusted analysis (odds ratio 0.91, 95% CI: 0.81–1.01, *p* = 0.08), although the pre-specified adjusted analysis demonstrated a significant difference in favour of continuous infusion (odds ratio 0.89, 95% CI: 0.81–0.99, *p* = 0.04). Test of cure at 14 days was significantly different (5.7% absolute difference in favour of continuous infusion, *p* < 0.001), while the other outcome measures did not meet the test of significance, point estimates all favoured continuous infusion. Based on the statistically non-significant 2% reduction in mortality at 90 days, the authors estimated a number needed to treat of 50 to save one additional life. A meta-analysis, conducted by the BLING III team,^5^ published simultaneously with the main trial manuscript found a significant benefit of continuous versus intermittent infusions in terms of 90-day mortality and clinical cure. The number needed to treat, estimated from the meta-analysis, was 25 to save one additional life at 90 days, with a 99.1% posterior probability of lower 90-day mortality. The accompanying editorial stated, ‘clinical guidelines are likely to use this new landmark study and accompanying meta-analysis to strengthen their recommendation to use continuous β-lactam antibiotics over intermittent dosing in adult patients with sepsis in the ICU’.^6^

The purpose of this secondary analysis is to present data from the UK contribution to the global trial and to allow a degree of comparison between the UK data and the global dataset of which it forms a significant part. We also report on adverse events and protocol violations, which might have a bearing on adoption of continuous infusion into practice. Finally, comprehensive and current data from a large and arguably representative group of patients is of value for planning future studies.

## Methods

The BLING III trial was an international open-label, randomised, non-blinded clinical trial of continuous versus intermittent infusion of beta-lactam antibiotics with the primary outcome of 90-day all-cause mortality.

In the UK, the Medicines and Healthcare products Regulatory Agency determined this was a trial of clinical protocols and not a clinical trial of an investigational medicinal product. The trial received approval from South Central – Berkshire Research Ethics Committee (18/SC/0242) and the Health Research Authority and was registered with ClinicalTrials.gov (NCT03213990). The UK sponsor was The George Institute for Global Health, via its UK office. Imperial College London delivered the trial along with the participating investigators and site teams. Funding was provided by the Australian National Health and Medical Research Council; the trial was a portfolio study supported by the National Institute for Health Research.

The global trial ran from 26th March 2018, until 11th January 2023, with 90-day follow up completed on 23rd April 2023. The study was disrupted by the SARS-CoV-2 (COVID-19) pandemic, as research resources pivoted towards pandemic-related research.

Written informed consent was provided by participants with capacity, for those without current capacity, legal or professional surrogates were consulted according to standard UK practice. Recovering participants were approached to confirm consent to continue.

Detailed trial methods have been published in the trial protocol paper^7^ and the main manuscript.^1^ In brief:

### Setting

The trial was conducted in 104 intensive care units worldwide and 54 in the UK (50 in England, 3 in Scotland and 1 in Wales).

### Participants

Adult patients (⩾18 years old) being treated in a critical care unit with a documented site or strong clinical suspicion of infection, organ dysfunction, and who had been started on meropenem or piperacillin-tazobactam within the previous 24 h. Participants were anticipated to be resident in the ICU for at least the next calendar day (see Supplemental 3 in the main manuscript^1^ for full inclusion and exclusion criteria). Key exclusion criteria were established renal failure, anticipated immediate requirement for renal replacement therapy and documented sensitivity to either drug.

### Randomisation

Participants were randomised to receive the beta-lactam antibiotic via continuous or intermittent infusion in a 1:1 ratio using a web-based secured system, with stratification by site only.

### Interventions

Drug, dose and treatment duration were determined by the clinical team. Participants received at least one treatment dose by short intermittent infusion, as a loading dose, prior to receiving drug by open-label study-allocated method. Administration method was either continuous infusion or short intermittent infusions with dose delivered over a 30-min period. Cross-over to an alternative beta-lactam antibiotic (meropenem or piperacillin-tazobactam) was permitted, however, the allocated administration method remained the same for the treatment course. Allocated treatment method remained in place for 14 days after randomisation or discharge from the ICU; if treatment was discontinued and subsequently restarted, the original allocated treatment method was used whilst the participant remained in the ICU. Detailed operating procedures are in Supplemental 3 of the main report.^1^

### Outcome measures

The primary outcome measure was 90-day all-cause mortality. Secondary outcome measures were test of clinical cure (defined as completion of the treatment course within 14 days without restarting antibiotics for the same infection within 48 h); acquisition of new colonisation and/or infection with multi-resistant organisms or *Clostridium difficile*, identified with routine sampling; and all cause ICU and hospital mortality. Tertiary outcomes were the number of days alive and free of ICU, hospital, ventilatory support and renal replacement therapy at 90 days after randomisation.

### Statistical analysis

The detailed statistical methods are published in the report of the global study and the statistical analysis plan.^1,8^ Analysis was based on a modified intention-to-treat analysis, using logistical regression with treatment allocation as a fixed effect and site as a random effect. Generally, results are presented as odds ratios (ORs) and 95% confidence intervals, converted to absolute differences (ADs) in proportions. There was a pre-specified analysis of the primary outcome with adjustment for APACHE II score at randomisation, sex, source of admission, and meropenem versus piperacillin-tazobactam. There were also pre-specified subgroup analyses for pre-randomisation factors: pulmonary infection, type of antibiotic, age (<65 or ⩾65 years), sex, and APACHE score (<25 or ⩾25). Other binary variables were analysed with logistic regression, and time-to-event analyses using cumulative incidence with death as a competing risk, censored at 90 days. Adverse events and protocol violations were explored with Fisher’s exact test.

The UK analysis followed the same statistical methods as for the global trial. The original sample size estimate was that 7000 participants would be required to detect an absolute difference of 3.5% in 90-day all-cause mortality with 90% power. This was developed from a baseline mortality rate of 27.5% and an alpha level of 0.05. Therefore, the UK sub-analysis is not intended to provide definitive answers to the main research questions, while providing important descriptive and comparative data. Nevertheless, as this is one of the largest trial cohorts in sepsis from the UK, it seemed important to make these data available, along with adverse event and protocol violation data.

## Results

**Figure 1.**
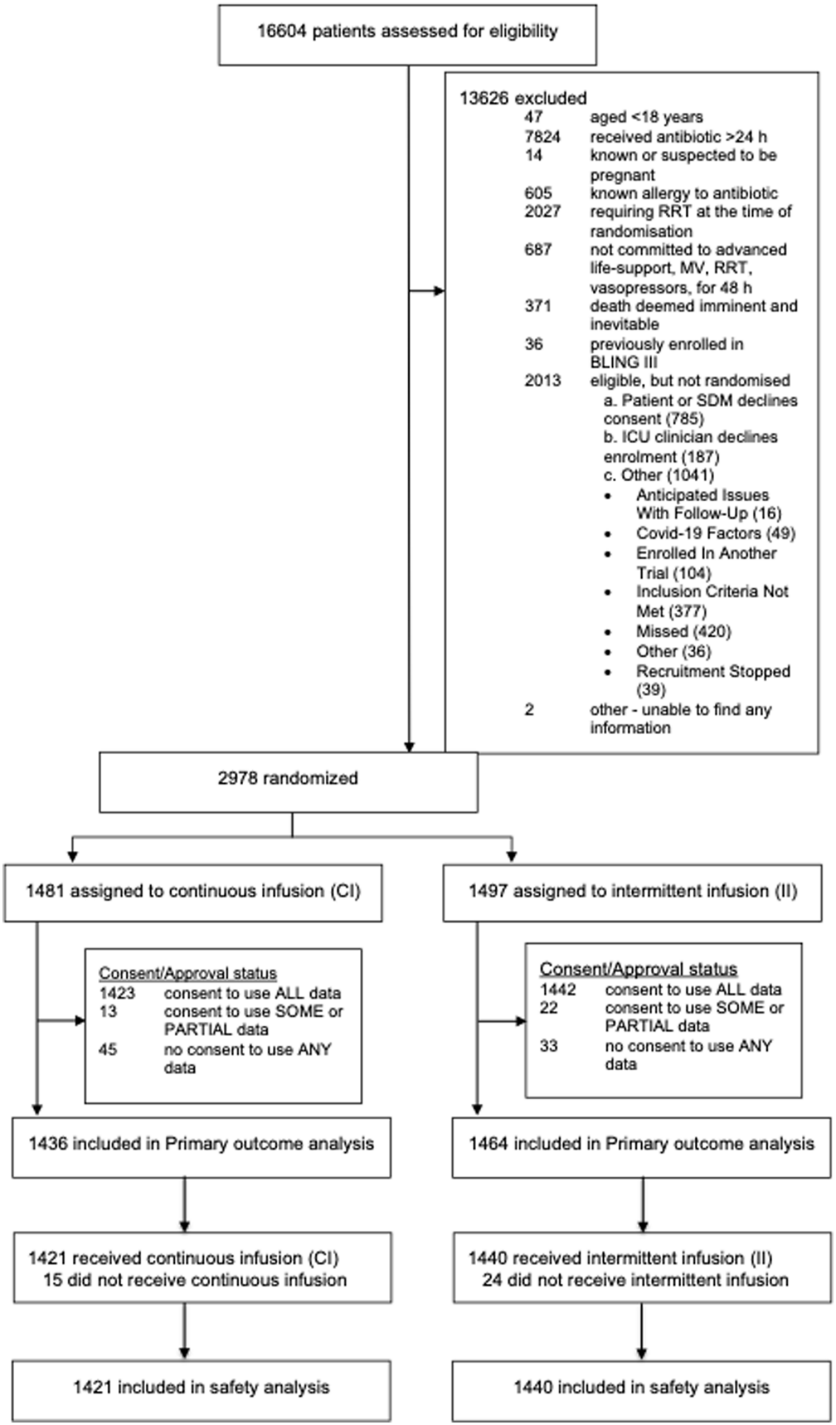
CONSORT flow diagram.

**Figure 2.**
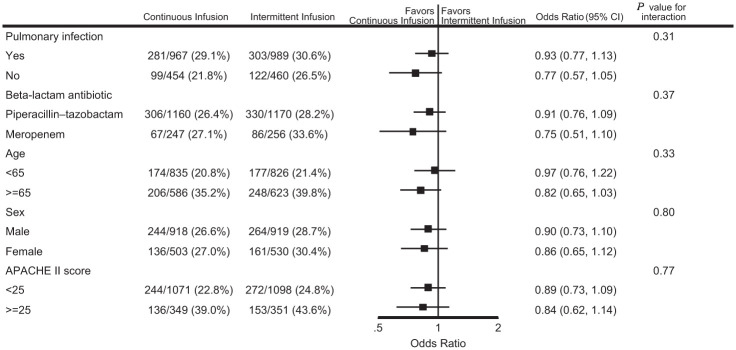
Forest plot for subgroup analysis of mortality at day 90. *Note*: Odds ratio estimated from logistic regression, with treatment allocation, subgroup variable, and the interaction between intervention and subgroup as a fixed effect as fixed effect, and site as a random effect.

**Figure 3.**
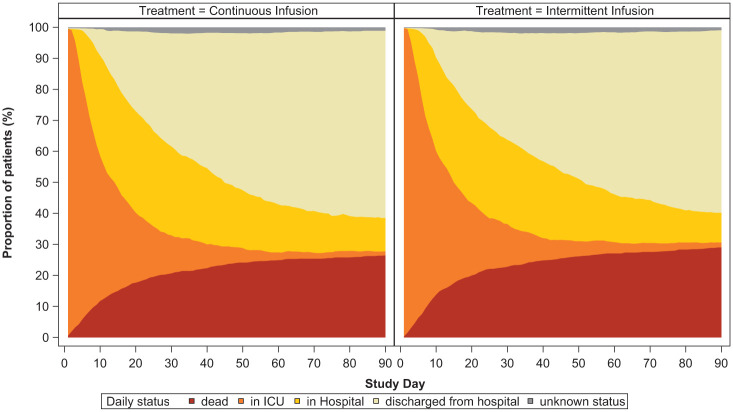
Daily participation disposition.

**Figure 4.**
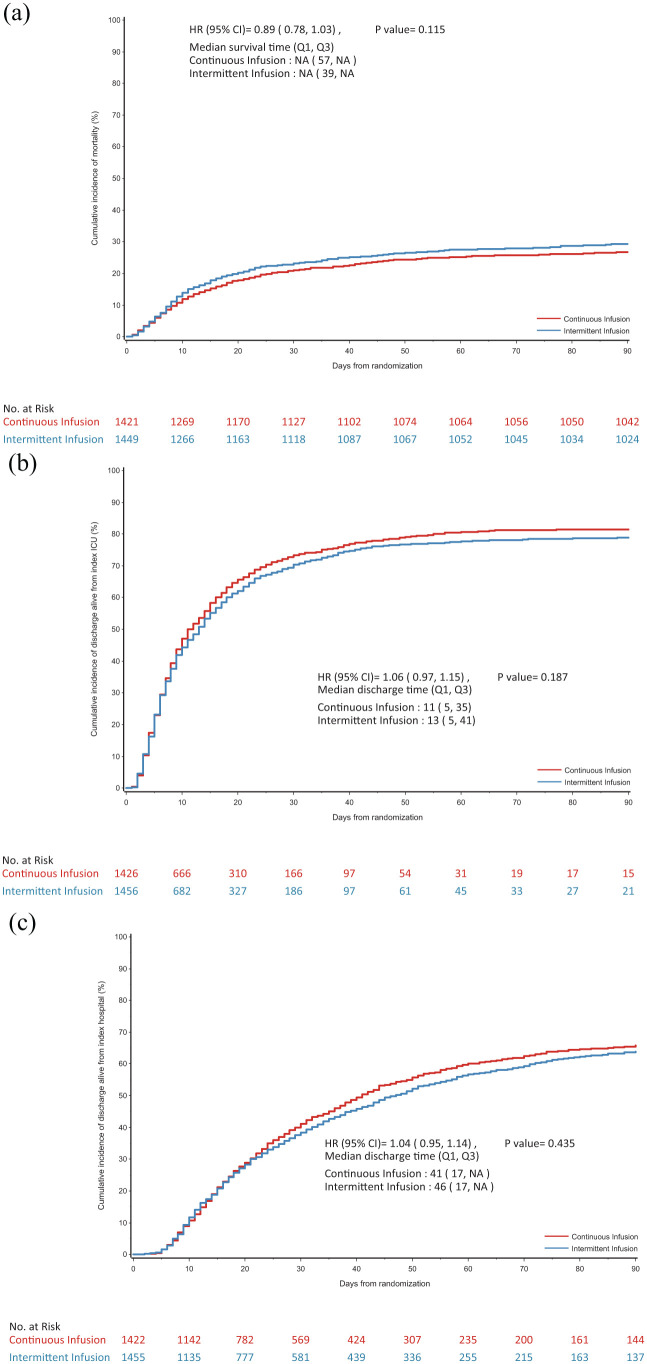
(a) Cumulative incidence function of time to death. (b) Cumulative incidence function of time to alive discharge from index ICU admission. (c) Cumulative incidence function of time to alive discharge from index hospital admission.

A total of 2900 participants from the UK were randomised and reported in the primary analysis. Participant baseline characteristics are reported in Table 1, with further characteristics presented in Supplemental Table 1 (with APACHE diagnostic data in Supplemental Tables 2 and 3); Figure 1 presents the CONSORT flow diagram for the UK cohort. The pooled 90-day mortality of the UK cohort was 28%, in line with the estimate used in the samples size calculation. Tables 2 and 3 displays the results for the main results; totals do not sum to 100%, as a small number of participants were lost to follow up. Generally, all comparisons favoured continuous infusion, however, these observations did not reach statistical significance. Overall, the results of the UK cohort are broadly consistent with the results reported in the global manuscript. For the primary outcome, the odds ratio favoured continuous infusions in the primary analysis (OR: 0.88, 95% CI: 0.75–1.04, *p* = 0.13) and adjusted analysis (OR: 0.87, 95% CI: 74–1.04, *p* = 0.12). Test of cure at day 14 also favoured continuous infusion, but did not achieve statistical significance (OR: 1.13, 95% CI: 0.97–1.13, *p* = 0.11). The detailed outcome analyses are presented in Tables 2 and 3, with the pre-specified subgroup analyses presented in Figure 2. Although point estimates in all analyses are in the direction of favouring continuous infusion, none met statistical significance. There appeared no impact on acquisition of resistant organisms. Figure 3 presents daily disposition of participants from randomisation to day 90, with time to event analyses presented in Figure 4.

**Table 1.** Baseline characteristics.

Characteristics	Continuous infusion (*N* = 1436)	Intermittent infusion (*N* = 1464)	Total (*N* = 2900)
Age (years) – *N*	1436	1464	2900
Mean (SD)	59.0 (15.84)	59.5 (15.50)	59.3 (15.67)
Gender – *N*	1436	1464	2900
Male	930 (64.8%)	932 (63.7%)	1862 (64.2%)
Female	506 (35.2%)	532 (36.3%)	1038 (35.8%)
Weight (kg) – *N*	1433	1463	2896
Mean (SD)	81.8 (21.76)	82.1 (21.18)	82.0 (21.47)
Height (cm) – *N*	1416	1458	2874
Mean (SD)	170.8 (10.01)	170.5 (9.81)	170.7 (9.90)
Source of ICU admission – *N*	1435	1464	2899
Accident and emergency department	486 (33.9%)	488 (33.3%)	974 (33.6%)
Hospital floor (i.e. wards)	480 (33.4%)	499 (34.1%)	979 (33.8%)
Transfer from another ICU	40 (2.8%)	44 (3.0%)	84 (2.9%)
Transfer from another hospital (except from another ICU)	38 (2.6%)	46 (3.1%)	84 (2.9%)
Admitted from operating theatre following emergency surgery	303 (21.1%)	303 (20.7%)	606 (20.9%)
Admitted from Operating Theatre following elective surgery	88 (6.1%)	84 (5.7%)	172 (5.9%)
Time from ICU admission to randomisation (h) – *N*	1435	1464	2899
Mean (SD)	95.3 (143.99)	94.7 (187.78)	95.0 (167.51)
Median (Q1; Q3)	44.8 (16.0; 120.7)	42.4 (15.1; 118.7)	43.4 (15.6; 120.3)
APACHE II score – *N*	1435	1464	2899
Mean (SD)	19.4 (7.52)	19.4 (7.42)	19.4 (7.47)
Median (Q1; Q3)	19.0 (14.0; 24.0)	19.0 (14.0; 24.0)	19.0 (14.0; 24.0)
Lowest PaO2/FIO2 ratio in the 24 h prior to randomisation – *N*	1338	1365	2703
Mean (SD)	182.4 (103.04)	181.4 (98.67)	181.9 (100.84)
Median (Q1; Q3)	159.4 (102.8; 240.0)	159.0 (105.0; 240.0)	159.0 (103.5; 240.0)
Highest creatinine (μmol/L) – *N*	1429	1458	2887
Mean (SD)	112.7 (90.48)	113.3 (98.88)	113.0 (94.80)
Median (Q1; Q3)	83.0 (58.0; 135.0)	80.5 (58.0; 132.0)	82.0 (58.0; 133.0)
Highest bilirubin (μmol/L) – *N*	1357	1392	2749
Mean (SD)	23.6 (42.49)	23.7 (49.06)	23.6 (45.92)
Median (Q1; Q3)	12.0 (8.0; 22.0)	12.0 (8.0; 21.0)	12.0 (8.0; 21.0)
Lowest platelet count (×10/L) – *N*	1426	1453	2879
Mean (SD)	241.8 (149.12)	237.6 (139.83)	239.7 (144.50)
Median (Q1; Q3)	213.0 (142.0; 308.0)	212.0 (146.0; 305.0)	213.0 (144.0; 307.0)
Lowest MAP in 24 h prior to randomisation (mmHg) – *N*	1430	1452	2882
Mean (SD)	66.0 (12.82)	65.5 (11.92)	65.8 (12.37)
Median (Q1; Q3)	64.0 (59.0; 71.0)	64.0 (59.0; 72.0)	64.0 (59.0; 71.0)
Worst Glasgow Coma Score (non-sedated) – *N*	1113	1127	2240
Mean (SD)	11.4 (4.55)	11.5 (4.32)	11.4 (4.43)
Median (Q1; Q3)	14.0 (8.0; 15.0)	14.0 (8.0; 15.0)	14.0 (8.0; 15.0)
Received inotropes/vasopressors in the 24 h prior to randomisation	954/1435 (66.5%)	960/1464 (65.6%)	1914/2899 (66.0%)
Received antibiotic(s) in the 24 h prior to randomisation*	908/1435 (63.3%)	989/1464 (67.6%)	1897/2899 (65.4%)

ICU: intensive care unit; APACHE: acute physiology and chronic health evaluation; PaO_2_: arterial partial pressure of oxygen; Fi0_2_: fraction of inspired oxygen; MAP: mean arterial pressure.

* Other than piperacillin–tazobactam or meropenem.

**Table 2.** Primary and secondary outcomes.

Outcome	Continuous infusion (*N* = 1436)	Intermittent infusion (*N* = 1464)	Total (*N* = 2900)	*P*-Chi^2^	Absolute difference (95% CI)	Odds ratio (95% CI)	*p*-Model
All-cause mortality by day 90	380/1421 (26.7%)	425/1449 (29.3%)	805/2870 (28.0%)	0.1227	−2.58 % (−6.56%, 1.41%)	0.88 (0.75, 1.04)	0.1265
All-cause mortality by day 90^a^					−2.64% (−7.24%, 1.97%)	0.87 (0.74, 1.03)	0.1163
Clinical cure at day 14^b^	833/1424 (58.5%)	802/1445 (55.5%)	1635/2869 (57.0%)	0.1052	3.00% (−1.39%, 7.40%)	1.13 (0.97, 1.31)	0.1056
Colonisation or infection with a MRO or clostridium difficile up to day 14	90/1436 (6.3%)	90/1464 (6.1%)	180/2900 (6.2%)	0.8936	0.14% (−2.02%, 2.30%)	1.03 (0.76, 1.39)	0.8720
All-cause ICU mortality by day 90	263/1421 (18.5%)	304/1449 (21.0%)	567/2870 (19.8%)	0.0963	−2.49% (−6.25%, 1.27%)	0.86 (0.71, 1.03)	0.0971
All-cause hospital mortality by day 90^c^	359/1421 (25.3%)	401/1449 (27.7%)	760/2870 (26.5%)	0.1434	−2.41% (−6.17%, 1.35%)	0.88 (0.75, 1.04)	0.1461

SD: standard deviation; OR: odds ratio; CI: confidence interval; ICU: intensive care unit; MRO: multiresistant organism.

Absolute difference (95% CI), odds ratio (95% CI) and *p*-model are estimated from logistic regression, with treatment allocation as a fixed effect and site as a random effect.

a Adjusted analyses are performed by adding the following covariates to the main logistic regression model: sex, Acute Physiology and Chronic Health Evaluation (APACHE) III score at randomisation (as a continuous variable),source of admission (admitted from the operating theatre following emergency or elective surgery vs other) and type of beta-lactam antibiotic administered (piperacillin–tazobactam or meropenem). Number of patients read: 2900, number of patients used: 2833, percentage of missing: 2.3%.

b Clinical cure is defined as the completion of the beta-lactam antibiotic treatment course on or before day 14 without recommencement of antibiotic therapy within 48 h of cessation for the same infectious episode.

c Patients died in ICU are also patients died in hospital; Missing data: Day 90 mortality: Number of patients lost to follow-up (18) or mortality status unknown (12); Day 14 clinical cure: 31. Colonisation or infection with a MRO or Clostridium difficile up to day 14: 0.

**Table 3.** Tertiary outcomes reported as days alive and free of the outcome 90 days post randomization.

Outcome	Continuous infusion (*N* = 1436)	Intermittent infusion (*N* = 1464)	Total (*N* = 2900)	Mean difference (95% CI)	*p*
ICU free days	1426	1456	2882	2.20 (−0.31, 4.71)	0.0856
Mean (SD)	56.1 (34.30)	53.9 (34.84)	55.0 (34.58)		
Median (Q1; Q3)	75.0 (17.0; 83.0)	72.0 (7.0; 83.0)	73.0 (11.0; 83.0)		
Hospital free days	1422	1455	2877	1.79 (−0.56, 4.14)	0.1353
Mean (SD)	37.8 (32.49)	36.0 (32.67)	36.9 (32.58)		
Median (Q1; Q3)	44.0 (0.0; 69.0)	38.0 (0.0; 69.0)	40.0 (0.0; 69.0)		
Days free of mechanical ventilation	1427	1457	2884	1.80 (−0.76, 4.36)	0.1683
Mean (SD)	62.1 (35.11)	60.3 (35.63)	61.2 (35.38)		
Median (Q1; Q3)	82.0 (29.0; 90.0)	81.0 (21.0; 89.0)	82.0 (23.0; 89.5)		
Days free of RRT	1430	1457	2887	1.94 (−0.51, 4.39)	0.1200
Mean (SD)	70.2 (33.21)	68.3 (34.28)	69.2 (33.76)		
Median (Q1; Q3)	90.0 (49.0; 90.0)	90.0 (37.0; 90.0)	90.0 (44.0; 90.0)		

SD: standard deviation; CI: confidence interval; ICU: intensive care unit; RRT: renal replacement therapy.

Duration is compared as days alive and free of the outcome between treatment groups using linear regression with a fixed effect of the treatment group and a random effect of site. No adjusted or subgroup analyses are applied. Number of patients without enough information to estimate: Follow-up duration: 13, Days free of ICU: 18, Days free of hospital: 23, Days free of mechanic ventilation: 16, Days free of renal replacement therapy: 13.

Table 4 summarises primary sites of infection which were dominated by pulmonary and abdominal infections (65.6% and 12.1%, respectively); these were well balanced and broadly in line with the global report. There seems a greater proportion of participants with a primary site of infection in their lungs in the UK data compared with the global report (59.5%). A plausible causative organism was identified in 28.7% of participants (Supplemental Table 5), the majority of which were gram negative (Escherichia, Klebsiella and Pseudomonas species); the dominant gram-positive organisms identified were Methicillin-sensitive *Staphylococcus aureus* or *Streptococcus pneumoniae*. The patterns were broadly similar to that reported in the global manuscript, although overall fewer primary causative organisms were identified (28.8% vs 40.8%). Data concerning second sites of infection and associated microbiology are presented in Supplemental Table 6.

**Table 4.** Sites of infection.

Details of infection	Continuous infusion (*N* = 1436)	Intermittent infusion (*N* = 1464)	Total (*N* = 2900)
Primary site of infection	*n* = 1435	*n* = 1464	*n* = 2899
Pulmonary	943 (65.7%)	958 (65.4%)	1901 (65.6%)
Intra-abdominal	172 (12.0%)	180 (12.3%)	352 (12.1%)
Blood	53 (3.7%)	56 (3.8%)	109 (3.8%)
Skin	61 (4.3%)	62 (4.2%)	123 (4.2%)
Urinary	64 (4.5%)	51 (3.5%)	115 (4.0%)
Intravenous catheter	7 (0.5%)	5 (0.3%)	12 (0.4%)
Central nervous system	41 (2.9%)	39 (2.7%)	80 (2.8%)
Gut	43 (3.0%)	65 (4.4%)	108 (3.7%)
Endocarditis	4 (0.3%)	1 (0.1%)	5 (0.2%)
Other	47 (3.3%)	47 (3.2%)	94 (3.2%)
Ear infection	0 (0.0%)	1 (0.1%)	1 (0.0%)
Gynaecological	1 (0.1%)	1 (0.1%)	2 (0.1%)
Infected graft/hardware	1 (0.1%)	0 (0.0%)	1 (0.0%)
Intra thoracal	6 (0.4%)	4 (0.3%)	10 (0.3%)
Musculoskeletal	11 (0.8%)	8 (0.5%)	19 (0.7%)
Oro naso pharyngal	5 (0.3%)	10 (0.7%)	15 (0.5%)
Soft tissue	5 (0.3%)	4 (0.3%)	9 (0.3%)
Unknown	18 (1.3%)	19 (1.3%)	37 (1.3%)
Second site of infection	*n* = 228	*n* = 229	*n* = 457
Pulmonary	75 (32.9%)	93 (40.6%)	168 (36.8%)
Intra-abdominal	31 (13.6%)	14 (6.1%)	45 (9.8%)
Blood	39 (17.1%)	39 (17.0%)	78 (17.1%)
Skin	24 (10.5%)	20 (8.7%)	44 (9.6%)
Urinary	18 (7.9%)	24 (10.5%)	42 (9.2%)
Intravenous catheter	9 (3.9%)	10 (4.4%)	19 (4.2%)
Central nervous system	12 (5.3%)	9 (3.9%)	21 (4.6%)
Gut	7 (3.1%)	9 (3.9%)	16 (3.5%)
Endocarditis	4 (1.8%)	1 (0.4%)	5 (1.1%)
Other	9 (3.9%)	10 (4.4%)	19 (4.2%)
Gynaecological	0 (0.0%)	1 (0.4%)	1 (0.2%)
Intra thoracal	2 (0.9%)	0 (0.0%)	2 (0.4%)
Musculoskeletal	2 (0.9%)	0 (0.0%)	2 (0.4%)
Oro naso pharyngal	2 (0.9%)	3 (1.3%)	5 (1.1%)
Soft tissue	1 (0.4%)	1 (0.4%)	2 (0.4%)
Unknown	2 (0.9%)	5 (2.2%)	7 (1.5%)
Third site of infection	*n* = 44	*n* = 38	*n* = 82
Pulmonary	15 (34.1%)	13 (34.2%)	28 (34.1%)
Intra-abdominal	5 (11.4%)	4 (10.5%)	9 (11.0%)
Blood	6 (13.6%)	8 (21.1%)	14 (17.1%)
Skin	14 (31.8%)	6 (15.8%)	20 (24.4%)
Urinary	4 (9.1%)	2 (5.3%)	6 (7.3%)
Intravenous catheter	0 (0.0%)	0 (0.0%)	0 (0.0%)
Central nervous system	0 (0.0%)	0 (0.0%)	0 (0.0%)
Gut	0 (0.0%)	2 (5.3%)	2 (2.4%)
Endocarditis	0 (0.0%)	0 (0.0%)	0 (0.0%)
Other	0 (0.0%)	3 (7.9%)	3 (3.7%)
Musculoskeletal	0 (0.0%)	2 (5.3%)	2 (2.4%)
Oro naso pharyngal	0 (0.0%)	1 (2.6%)	1 (1.2%)

There were very few adverse events (Supplemental Table 7) equally distributed between treatment groups. Protocol deviations are presented in Table 5; overall 36.8% of participants experienced a protocol deviation, the vast majority of which were related to dosing and administration. Participants often received multiple violations.

**Table 5.** Summary of protocol deviations.

Protocol deviation	Continuous infusion (*N* = 1421)	Intermittent infusion (*N* = 1440)	Total (*N* = 2861)	*p*-Value
Any protocol deviation	1650^a^ 538 (37.9%)	1277^a^ 515 (35.8%)	2927^a^ 1053 (36.8%)	0.2609
Randomisation of ineligible patient	62^a^ 62 (4.4%)	65^a^ 65 (4.5%)	127^a^ 127 (4.4%)	
No dose prior to randomisation	22 22 (1.5%)	30 30 (2.1%)	52 52 (1.8%)	
>24 h of BL	27 27 (1.9%)	21 21 (1.5%)	48 48 (1.7%)	
Other	8 8 (0.6%)	9 9 (0.6%)	17 17 (0.6%)	
Allergy	5 5 (0.4%)	5 5 (0.3%)	10 10 (0.3%)	
Follow up assessment not done correctly	32^a^ 32 (2.3%)	28^a^ 28 (1.9%)	60^a^ 60 (2.1%)	
Total number of doses given	*n* = 21,242	*n* = 24,806	*n* = 46,048	
Betalactam dosing related PDs	1540^a^ 470 (33.1%)	1172^a^ 441 (30.6%)	2712^a^ 911 (31.8%)	
Drug given/started early	55 54 (3.8%)	2 2 (0.1%)	57 56 (2.0%)	
No bolus/loading dose	51 48 (3.4%)	1 1 (0.1%)	52 49 (1.7%)	
Dose preparation incorrect – that is, wrong solution	4 4 (0.3%)	0 0 (0.0%)	4 4 (0.1%)	
Meropenem > 8 h (for interest/allowed)	2 2 (0.1%)	0 0 (0.0%)	2 2 (0.1%)	
Pause > 1 h in continuous arm/delay > 1 h in intermittent arm	385^a^ 260 (18.3%)	546^a^ 320 (22.2%)	931^a^ 580 (20.3%)	
Clinician decision to not give via study assigned administration method	106^a^ 9 (0.6%)	7^a^ 4 (0.3%)	113^a^ 13 (0.5%)	
Overdosed	1^a^ 1 (0.1%)	1^a^ 1 (0.1%)	2^a^ 2 (0.1%)	
Underdosed	1^a^ 1 (0.1%)	0^a^ 0 (0.0%)	1^a^ 1 (0.0%)	
Intermittent dose not given as 30 min infusion	31^a^ 7 (0.5%)	200^a^ 57 (4.0%)	231^a^ 64 (2.2%)	
Missed dose	37^a^ 31 (2.2%)	43^a^ 38 (2.6%)	80^a^ 69 (2.4%)	
Delayed infusion start/dose start	20^a^ 19 (1.3%)	2^a^ 2 (0.1%)	22^a^ 21 (0.7%)	
Incorrect study administration method used in error	807^a^ 147 (10.3%)	364^a^ 70 (4.9%)	1171^a^ 217 (7.6%)	
Reason not given via study assigned administration method – other	40^a^ 11 (0.8%)	6^a^ 5 (0.3%)	46^a^ 16 (0.6%)	
Other	16^a^ 16 (1.1%)	12^a^ 12 (0.8%)	28^a^ 28 (1.0%)	
Day 90 follow up for EQ	3 3 (0.2%)	8 8 (0.6%)	11 11 (0.4%)	
Allowed within the protocol	6 6 (0.4%)	2 2 (0.1%)	8 8 (0.3%)	
Hospital error dosing within protocol/randomised pre-consent	6 6 (0.4%)	2 2 (0.1%)	8 8 (0.3%)	
Other	1 1 (0.1%)	0 0 (0.0%)	1 1 (0.0%)	
Consent	1 1 (0.1%)	0 0 (0.0%)	1 1 (0.0%)	

The denominators for percentages are all randomised patients- *p*-values by Fisher exact test.

a PDs are summarised as the total number of violations reported, followed by the number and percentage of patients having at least one violation.

Additional antibiotics were prescribed during the study period for 80.4% of participants and are presented in Supplemental Table 8; this includes co-prescription alongside the beta-lactam antibiotic (study drug) and ‘step down’ antibiotics after microbiology results had been returned. On initial inspection, there seem some small differences between the UK and global cohorts (Dulhunty et al.^1^; Table 9), but having grouped vancomycin, teicoplanin and linezolid, and amikacin with gentamicin together, these small differences seemed much less obvious.

Investigators were asked to identify causes of death from a pre-specified list (rather than from the Medical Certificate of Cause of Death), this and place of death are presented in Supplemental Table 9. Compared to the global trial, place of death seemed similar, although fewer people from the UK cohort were described as dying from ‘Distributive (septic) shock’, with the commonest cause being ‘Hypoxic Respiratory Failure’.

Supplemental Table 10 presents the reason the beta-lactam antibiotic was stopped. This shows a highly significant difference between continuous and intermittent infusion for both courses completed and change in antibiotic therapy (Fisher’s exact test *p* < 0.001 for both comparisons).

The time interval between ICU admission and randomisation is different between the UK and the global report: a median of 43.4 h in the UK cohort compared with 25 h in the global report, suggesting greater numbers of patients already in ICU. The distributions of time intervals were subsequently examined and are presented in a Supplemental Figure. This comparison of UK versus non-UK data (rather than the total cohort) shows respective medians of 36.8 (IQR: 14.9–107.6) versus 20.2 (IQR: 10.1–77.6) hours (Mann–Whitney, *p* < 0.001).

## Discussion

The UK provided the largest single national contribution to BLING III trial, and comes from a single healthcare system which, arguably, has less treatment and process heterogeneity than the non-UK contribution. Given the anticipated effect size from which the trial was built, it was never likely that the UK cohort on its own would show a statistically significant difference between the two arms of the trial. That said, it is reassuring that all the point estimates remain consistent with the global trial and therefore the findings of the main BLING III trial and the contemporaneous meta-analysis are relevant to UK practice. Overall, the UK cohort is very similar to the global study population.

As the UK data are embedded within the global cohort, we must be careful drawing inferences. Any apparent differences that we may see between the two datasets might seem smaller as the presence of the UK *within* the global data will have the effect of bringing any estimate of central tendency together. As the remaining global data come from Australasia, South-East Asia and Europe we elected not to conduct a formal comparison between the UK and non-UK data as this would have been post hoc and would not address any of the objectives set out.

There are some observations that seem to stand out between the UK and the global datasets. The UK centres seemed to randomise patients somewhat later after ICU admission (Supplemental Figure). This raises the possibility that greater numbers of participants had been in ICU for some time, and who had a hospital or ICU acquired infection, rather than being admitted de novo with a severe infection. Other possibilities are that in the UK decisions to initiate antibiotic treatment were slower or perhaps, more likely, initial treatment did not include meropenem or piperacillin-tazobactam. It seems unlikely that this was due to delays in acquiring consent as a deferred consent model was used in the UK.

In addition, in the UK data there were fewer positive identifications of causative organisms compared with the overall dataset. The most common primary site of causative infection in both data sets was pulmonary (approximately 60%). It is not clear why fewer culprit organisms were identified in the UK; 28.7% c.f. 40.8% for primary infection, although the pattern of organisms identified is similar. It seems unlikely, although plausible, that this reflects pre-trial antibiotic use interfering with laboratory culture performance; it is a reasonable assumption that all of the participating hospitals will have been operating similar timeline-driven approaches to initial management of severe infection, as per national and international sepsis guidelines.

There were very few adverse events reported. In the UK data, only 11 adverse events occurred and none were serious. Acquisition of C. difficile and other resistant organisms was a secondary outcome and was not different between groups. The numbers and types of protocol violations might give some insight into bedside practical challenges of differences in mode of delivery. There was a very large number of individual protocol violations with 32% of participants experiencing a dosing or administration error. This was marginally more common in the continuous infusion arm for ‘incorrect administration method used’ and often occurred multiple times per participant. Both groups also experienced pauses and delays in infusion, despite detailed instructions on how to manage pauses for interventions etc. This could represent an issue with training or organisational stress at the time; any implementation plan would need to address this.

It is not obvious why there was a clear difference in reasons for stopping the original trial prescription, also visible in the global report (Supplemental 3, Table 8).^1^ In the intermittent infusion group it was a fairly even split between course completion and change of prescription, while in the continuous infusion group its was more frequently due to change in therapy. In the global report, in both treatment arms a change in therapy was more common than completion of course, again with this much more likely in the continuous infusion arm. There seem several possible explanations, including random chance; loss of confidence in the current treatment plan; changing from broad to narrower spectrum, having received microbiology data and being content with patient progress with the test of cure data supporting the latter assertion.

Taken together, these data provide reassurance that the key findings of the BLING III trial are applicable to UK practice. The rate of protocol violations, not all of which were to do with trial procedures, emphasises the need for a planned and education-based introduction of continuous infusion to those centres that choose to go this way.^9^ Some important questions remain unanswered. We remain unsure how far we can apply these findings to patients with established renal failure and how far we can extend these findings to other beta-lactam antibiotics. In addition, the study included the COVID-19 pandemic, with recruitment paused in some regions and centres. At least in part because of the pandemic, many intensive care services lost significant numbers of established nursing staff and have been rebuilding their workforce. This emphasises the need for careful planning and support for practice changes.

The overall detailed description, including microbiology, of a large cohort with severe infection/sepsis may have utility for future trial design, bearing in mind the exclusion for people requiring or anticipated to require, renal replacement therapy.

How we regard a comparison of these UK data with the global cohort of which they form part is an interesting question. The global cohort represents a wide variety of hospitals and countries. We have not chosen to compare the UK cohort with the non-UK remainder as we were not clear what purpose that would serve; it would not have addressed the question of applicability which we set ourselves. We did directly examine the distribution of time intervals between admission and randomisation, but this was looking for major differences in patient location prior to the index episode of infection, that is, were more patients *already resident* in the ICU when they developed an infection, rather than being admitted *with* a severe infection. Unfortunately, we recorded time and source of admission, but we did not record the actual location of patients at the time they developed their index infection, so this could not be explored further.

## Conclusion

Bling III was an international trial comparing bolus dosing and continuous infusions of piperacillin-tazobactam and meropenem in 104 ICUs from 7 countries of which 54 of the ICUs were in the UK. In this manuscript we present the data from these 54 ICUs with the relevant comparisons of the two groups (bolus dosing vs continuous infusions) in the Tables attached. Data and results presented here were similar to the overall trial results and hence there seems to be no reason why the results of the global BLING III trial do not apply to the UK setting.

## Supplemental Material

sj-docx-1-inc-10.1177_17511437251396871 – Supplemental material for Continuous versus intermittent beta-lactam antibiotic infusions in critically ill patients: The UK cohort of the BLING III trial Supplemental material, sj-docx-1-inc-10.1177_17511437251396871 for Continuous versus intermittent beta-lactam antibiotic infusions in critically ill patients: The UK cohort of the BLING III trial by Janis Best-Lane, Farah Al-Beidh, Greg Barton, Dorrilyn Rajbhandari, Xiaoqiu Liu, Jayanthi Mysore, Serena Knowles, Naomi Hammond, Joel Dulhunty, John Myburgh, Jeffrey Lipman and Stephen J. Brett in Journal of the Intensive Care Society
