# Supplementary material for: Continuous versus intermittent beta-lactam antibiotic infusions in critically ill patients: The UK cohort of the BLING III trial
Source: J Intensive Care Soc. 2025 Nov 27;27(1):30–40. doi: 10.1177/17511437251396871 (PMC12662772; doi:10.1177/17511437251396871)
Supplement: sj-docx-1-inc-10.1177_17511437251396871 – Supplemental material for Continuous versus intermittent beta-lactam antibiotic infusions in critically ill patients: The UK cohort of the BLING III trial [file sj-docx-1-inc-10.1177_17511437251396871.docx]

**Supplementary Table 1: BASELINE CHARACTERISTICS – with full descriptive characteristics**

| **Characteristics** | **Continuous Infusion (N = 1436)** | **Intermittent Infusion (N = 1464)** | **Total (N = 2900)** |
| --- | --- | --- | --- |
|  | | | |
| **Age (years)** |  |  |  |
| n | 1436 | 1464 | 2900 |
| Mean (SD) | 59.0 (15.84) | 59.5 (15.50) | 59.3 (15.67) |
| Median (Q1; Q3) | 61.0 (48.0; 72.0) | 62.0 (50.0; 71.0) | 62.0 (49.0; 71.0) |
| min max | 18 91 | 18 98 | 18 98 |
| P10; P90 | 37; 78 | 37; 77 | 37; 78 |
| **Age < 35** | 125/1436 (8.7%) | 126/1464 (8.6%) | 251/2900 (8.7%) |
| **Age >= 80** | 102/1436 (7.1%) | 98/1464 (6.7%) | 200/2900 (6.9%) |
|  | | | |
|  | | | |
| **Gender** |  |  |  |
| Male | 930/1436 (64.8%) | 932/1464 (63.7%) | 1862/2900 (64.2%) |
| Female | 506/1436 (35.2%) | 532/1464 (36.3%) | 1038/2900 (35.8%) |
|  | | | |
|  | | | |
| **Weight (kg)** |  |  |  |
| n | 1433 | 1463 | 2896 |
| Mean (SD) | 81.8 (21.76) | 82.1 (21.18) | 82.0 (21.47) |
| Median (Q1; Q3) | 78.0 (68.0; 92.0) | 80.0 (68.0; 92.0) | 79.0 (68.0; 92.0) |
| min max | 34 190 | 38 223 | 34 223 |
| P10; P90 | 58; 110 | 59; 109 | 59; 110 |
| **Weight < 60** | 153/1433 (10.7%) | 150/1463 (10.3%) | 303/2896 (10.5%) |
| **Weight >= 110** | 153/1433 (10.7%) | 143/1463 (9.8%) | 296/2896 (10.2%) |
|  | | | |
|  | | | |
| **Height (cm)** |  |  |  |
| n | 1416 | 1458 | 2874 |
| Mean (SD) | 170.8 (10.01) | 170.5 (9.81) | 170.7 (9.90) |
| Median (Q1; Q3) | 171.0 (165.0; 178.0) | 170.0 (164.0; 178.0) | 170.0 (164.0; 178.0) |
| min max | 137 200 | 115 203 | 115 203 |
| P10; P90 | 158; 183 | 158; 182 | 158; 183 |
| **Height < 160** | 172/1416 (12.1%) | 184/1458 (12.6%) | 356/2874 (12.4%) |
| **Height >= 185** | 118/1416 (8.3%) | 78/1458 (5.3%) | 196/2874 (6.8%) |
| **Height >= 190** | 32/1416 (2.3%) | 25/1458 (1.7%) | 57/2874 (2.0%) |
|  | | | |
|  | | | |
| **Source of ICU admission** |  |  |  |
| Accident and Emergency Department | 486/1435 (33.9%) | 488/1464 (33.3%) | 974/2899 (33.6%) |
| Hospital Floor (i.e. wards) | 480/1435 (33.4%) | 499/1464 (34.1%) | 979/2899 (33.8%) |
| Transfer from another ICU | 40/1435 (2.8%) | 44/1464 (3.0%) | 84/2899 (2.9%) |
| Transfer from another hospital (except from another ICU) | 38/1435 (2.6%) | 46/1464 (3.1%) | 84/2899 (2.9%) |
| Admitted from Operating Theatre following EMERGENCY surgery | 303/1435 (21.1%) | 303/1464 (20.7%) | 606/2899 (20.9%) |
| Admitted from Operating Theatre following ELECTIVE surgery | 88/1435 (6.1%) | 84/1464 (5.7%) | 172/2899 (5.9%) |
|  | | | |
|  | | | |
| **Time from ICU admission to randomisation (hours)** |  |  |  |
| n | 1435 | 1464 | 2899 |
| Mean (SD) | 95.3 (143.99) | 94.7 (187.78) | 95.0 (167.51) |
| Median (Q1; Q3) | 44.8 (16.0; 120.7) | 42.4 (15.1; 118.7) | 43.4 (15.6; 120.3) |
| min max | 0 1374 | 0 4351 | 0 4351 |
|  | | | |
|  | | | |
| **APACHE II score** |  |  |  |
| n | 1435 | 1464 | 2899 |
| Mean (SD) | 19.4 (7.52) | 19.4 (7.42) | 19.4 (7.47) |
| Median (Q1; Q3) | 19.0 (14.0; 24.0) | 19.0 (14.0; 24.0) | 19.0 (14.0; 24.0) |
| min max | 0 59 | 2 48 | 0 59 |
| P10; P90 | 10; 29 | 10; 29 | 10; 29 |
| **APACHE II score < 10** | 113/1435 (7.9%) | 116/1464 (7.9%) | 229/2899 (7.9%) |
| **APACHE II score >= 30** | 134/1435 (9.3%) | 132/1464 (9.0%) | 266/2899 (9.2%) |
|  | | | |
|  | | | |
| **Lowest PaO2/FIO2 ratio in the 24 hours prior to randomisation** |  |  |  |
| n | 1338 | 1365 | 2703 |
| Mean (SD) | 182.4 (103.04) | 181.4 (98.67) | 181.9 (100.84) |
| Median (Q1; Q3) | 159.4 (102.8; 240.0) | 159.0 (105.0; 240.0) | 159.0 (103.5; 240.0) |
| min max | 5 675 | 20 615 | 5 675 |
| P10; P90 | 72; 323 | 73; 326 | 73; 323 |
| **Lowest PaO2/FIO2 ratio < 100** | 321/1338 (24.0%) | 308/1365 (22.6%) | 629/2703 (23.3%) |
| **Lowest PaO2/FIO2 ratio >= 300** | 186/1338 (13.9%) | 199/1365 (14.6%) | 385/2703 (14.2%) |
|  | | | |
|  | | | |
| **Highest creatinine (umol/L)** |  |  |  |
| n | 1429 | 1458 | 2887 |
| Mean (SD) | 112.7 (90.48) | 113.3 (98.88) | 113.0 (94.80) |
| Median (Q1; Q3) | 83.0 (58.0; 135.0) | 80.5 (58.0; 132.0) | 82.0 (58.0; 133.0) |
| min max | 15 1173 | 5 1418 | 5 1418 |
| P10; P90 | 45; 219 | 46; 214 | 45; 216 |
| **Creatinine < 50** | 210/1429 (14.7%) | 213/1458 (14.6%) | 423/2887 (14.7%) |
| **Creatinine >= 300** | 66/1429 (4.6%) | 66/1458 (4.5%) | 132/2887 (4.6%) |
|  | | | |
|  | | | |
| **Highest bilirubin (umol/L)** |  |  |  |
| n | 1357 | 1392 | 2749 |
| Mean (SD) | 23.6 (42.49) | 23.7 (49.06) | 23.6 (45.92) |
| Median (Q1; Q3) | 12.0 (8.0; 22.0) | 12.0 (8.0; 21.0) | 12.0 (8.0; 21.0) |
| min max | 2 478 | 2 795 | 2 795 |
| P10; P90 | 5; 46 | 5; 43 | 5; 45 |
| **Bilirubin < 10** | 490/1357 (36.1%) | 500/1392 (35.9%) | 990/2749 (36.0%) |
| **Bilirubin >= 50** | 124/1357 (9.1%) | 114/1392 (8.2%) | 238/2749 (8.7%) |
|  | | | |
|  | | | |
| **Lowest platelet count (x10/L)** |  |  |  |
| n | 1426 | 1453 | 2879 |
| Mean (SD) | 241.8 (149.12) | 237.6 (139.83) | 239.7 (144.50) |
| Median (Q1; Q3) | 213.0 (142.0; 308.0) | 212.0 (146.0; 305.0) | 213.0 (144.0; 307.0) |
| min max | 1 1472 | 6 1362 | 1 1472 |
| P10; P90 | 89; 424 | 86; 404 | 88; 416 |
| **Platelet < 100** | 177/1426 (12.4%) | 183/1453 (12.6%) | 360/2879 (12.5%) |
| **Platelet >= 400** | 170/1426 (11.9%) | 158/1453 (10.9%) | 328/2879 (11.4%) |
|  | | | |
|  | | | |
| **Lowest MAP in 24 hours prior to randomisation (mmHg)** |  |  |  |
| n | 1430 | 1452 | 2882 |
| Mean (SD) | 66.0 (12.82) | 65.5 (11.92) | 65.8 (12.37) |
| Median (Q1; Q3) | 64.0 (59.0; 71.0) | 64.0 (59.0; 72.0) | 64.0 (59.0; 71.0) |
| min max | 28 158 | 28 121 | 28 158 |
| P10; P90 | 52; 81 | 53; 80 | 52; 81 |
| **MAP < 50** | 92/1430 (6.4%) | 87/1452 (6.0%) | 179/2882 (6.2%) |
| **MAP >= 80** | 187/1430 (13.1%) | 157/1452 (10.8%) | 344/2882 (11.9%) |
|  | | | |
|  | | | |
| **Worst Glasgow Coma Score (non-sedated)** |  |  |  |
| n | 1113 | 1127 | 2240 |
| Mean (SD) | 11.4 (4.55) | 11.5 (4.32) | 11.4 (4.43) |
| Median (Q1; Q3) | 14.0 (8.0; 15.0) | 14.0 (8.0; 15.0) | 14.0 (8.0; 15.0) |
| min max | 3 15 | 3 15 | 3 15 |
| P10; P90 | 3; 15 | 3; 15 | 3; 15 |
| **WGC Score < 6** | 206/1113 (18.5%) | 175/1127 (15.5%) | 381/2240 (17.0%) |
| **WGC Score = 15** | 485/1113 (43.6%) | 467/1127 (41.4%) | 952/2240 (42.5%) |
|  | | | |
|  | | | |
| **Received Inotropes/Vasopressors in the 24 hours prior to randomisation** | 954/1435 (66.5%) | 960/1464 (65.6%) | 1914/2899 (66.0%) |
|  | | | |
|  | | | |
| **Received antibiotic(s) in the 24 hours prior to randomisation*** | 908/1435 (63.3%) | 989/1464 (67.6%) | 1897/2899 (65.4%) |
|  | | | |

**Table Footnote**

*ICU = Intensive Care Unit,
APACHE = Acute Physiology and Chronic Health Evaluation,
PaO_2_ = Arterial Partial pressure of Oxygen,
Fi0_2_ = Fraction of inspired Oxygen,
MAP = Mean Arterial Pressure,
* Other than piperacillin–tazobactam or meropenem.*

**Supplementary Table 2: Top 10 Acute Physiology and Chronic Health Evaluation (APACHE) III diagnosis**

| **Characteristics** | **Continuous Infusion (N = 1436)** | **Intermittent Infusion (N = 1464)** | **Total (N = 2900)** |
| --- | --- | --- | --- |
|  | | | |
| **APACHE: Non-Operative Diagnosis** | 1044/1435 (72.8%) | 1077/1464 (73.6%) | 2121/2899 (73.2%) |
| 212 Bacterial Pneumonia | 121/1044 (11.6%) | 126/1077 (11.7%) | 247/2121 (11.6%) |
| 211 Other Respiratory Diseases | 77/1044 (7.4%) | 95/1077 (8.8%) | 172/2121 (8.1%) |
| 102 Cardiac Arrest | 87/1044 (8.3%) | 75/1077 (7.0%) | 162/2121 (7.6%) |
| 213 Viral Pneumonia | 71/1044 (6.8%) | 69/1077 (6.4%) | 140/2121 (6.6%) |
| 501 Sepsis other than urinary | 58/1044 (5.6%) | 73/1077 (6.8%) | 131/2121 (6.2%) |
| 601 Head Trauma +/- multi trauma | 58/1044 (5.6%) | 59/1077 (5.5%) | 117/2121 (5.5%) |
| 503 Sepsis with shock other than urinary tract | 47/1044 (4.5%) | 53/1077 (4.9%) | 100/2121 (4.7%) |
| 201 Aspiration Pneumonia | 41/1044 (3.9%) | 47/1077 (4.4%) | 88/2121 (4.1%) |
| 602 Multiple trauma excluding head | 46/1044 (4.4%) | 29/1077 (2.7%) | 75/2121 (3.5%) |
| 504 Sepsis with shock of urinary tract origin | 18/1044 (1.7%) | 9/1077 (0.8%) | 27/2121 (1.3%) |
| Other* | 420/1044 (40.2%) | 442/1077 (41.0%) | 862/2121 (40.6%) |
|  | | | |
|  | | | |
| **APACHE: Operative Diagnosis** | 391/1435 (27.2%) | 387/1464 (26.4%) | 778/2899 (26.8%) |
| 1401 GI perforation/rupture (not peritonitis) | 43/391 (11.0%) | 39/387 (10.1%) | 82/778 (10.5%) |
| 1412 Peritonitis | 31/391 (7.9%) | 40/387 (10.3%) | 71/778 (9.1%) |
| 1404 GI obstruction | 31/391 (7.9%) | 29/387 (7.5%) | 60/778 (7.7%) |
| 1405 GI Neoplasm | 19/391 (4.9%) | 25/387 (6.5%) | 44/778 (5.7%) |
| 1503 Subarachnoid haemorrhage | 20/391 (5.1%) | 23/387 (5.9%) | 43/778 (5.5%) |
| 1408 Other GI disease | 26/391 (6.6%) | 16/387 (4.1%) | 42/778 (5.4%) |
| 1904 Cellulitis / soft tissue infection | 17/391 (4.3%) | 18/387 (4.7%) | 35/778 (4.5%) |
| 1601 Head Trauma +/- Multi Trauma | 17/391 (4.3%) | 17/387 (4.4%) | 34/778 (4.4%) |
| 1602 Multiple Trauma excluding head | 12/391 (3.1%) | 11/387 (2.8%) | 23/778 (3.0%) |
| 1207 Coronary artery bypass grafts | 5/391 (1.3%) | 3/387 (0.8%) | 8/778 (1.0%) |
| Other* | 170/391 (43.5%) | 166/387 (42.9%) | 336/778 (43.2%) |
|  | | | |

**Table Footn*ote***

** Only the top 10 APACHE Non-operative and Operative diagnoses have been displayed with the rest in 'Other' category.
The denominators are those with any baseline APACHE data available.*

**Supplementary Table 3: Top 10 Acute Physiology and Chronic Health Evaluation (APACHE) III diagnosis- organ catagories**

| **Characteristics** | **Continuous Infusion (N = 1436)** | **Intermittent Infusion (N = 1464)** | **Total (N = 2900)** |
| --- | --- | --- | --- |
|  | | | |
| **APACHE: Non-Operative Diagnosis** | 1044/1435 (72.8%) | 1077/1464 (73.6%) | 2121/2899 (73.2%) |
| Cardiovascular | 113/1044 (10.8%) | 106/1077 (9.8%) | 219/2121 (10.3%) |
| Respiratory | 359/1044 (34.4%) | 392/1077 (36.4%) | 751/2121 (35.4%) |
| Gastrointestinal | 94/1044 (9.0%) | 90/1077 (8.4%) | 184/2121 (8.7%) |
| Neurological | 134/1044 (12.8%) | 133/1077 (12.3%) | 267/2121 (12.6%) |
| Sepsis | 150/1044 (14.4%) | 165/1077 (15.3%) | 315/2121 (14.9%) |
| Trauma | 126/1044 (12.1%) | 104/1077 (9.7%) | 230/2121 (10.8%) |
| Metabolic | 32/1044 (3.1%) | 25/1077 (2.3%) | 57/2121 (2.7%) |
| Haematological | 4/1044 (0.4%) | 6/1077 (0.6%) | 10/2121 (0.5%) |
| Renal/genitourinary | 6/1044 (0.6%) | 6/1077 (0.6%) | 12/2121 (0.6%) |
| Musculoskeletal/skin | 9/1044 (0.9%) | 15/1077 (1.4%) | 24/2121 (1.1%) |
| Other | 17/1044 (1.6%) | 35/1077 (3.2%) | 52/2121 (2.5%) |
|  | | | |
|  | | | |
| **APACHE: Operative Diagnosis** | 391/1435 (27.2%) | 387/1464 (26.4%) | 778/2899 (26.8%) |
| Cardiovascular | 32/391 (8.2%) | 35/387 (9.0%) | 67/778 (8.6%) |
| Respiratory | 12/391 (3.1%) | 16/387 (4.1%) | 28/778 (3.6%) |
| Gastrointestinal | 183/391 (46.8%) | 185/387 (47.8%) | 368/778 (47.3%) |
| Neurological | 80/391 (20.5%) | 79/387 (20.4%) | 159/778 (20.4%) |
| Trauma | 36/391 (9.2%) | 33/387 (8.5%) | 69/778 (8.9%) |
| Renal/genitourinary | 8/391 (2.0%) | 5/387 (1.3%) | 13/778 (1.7%) |
| Gynaecological | 4/391 (1.0%) | 1/387 (0.3%) | 5/778 (0.6%) |
| Musculoskeletal/skin | 35/391 (9.0%) | 30/387 (7.8%) | 65/778 (8.4%) |
| Haematological | 1/391 (0.3%) | 2/387 (0.5%) | 3/778 (0.4%) |
| Metabolic | 0/391 (0.0%) | 1/387 (0.3%) | 1/778 (0.1%) |
|  | | | |

**Table Footn*ote***

*The denominators are those with any baseline APACHE data available.*

**Supplementary Table 4**

| **Second site of infection** | **n=228** | **n=229** | **n=457** |
| --- | --- | --- | --- |
| Pulmonary | 75 (32.9%) | 93 (40.6%) | 168 (36.8%) |
| Intra-abdominal | 31 (13.6%) | 14 (6.1%) | 45 (9.8%) |
| Blood | 39 (17.1%) | 39 (17.0%) | 78 (17.1%) |
| Skin | 24 (10.5%) | 20 (8.7%) | 44 (9.6%) |
| Urinary | 18 (7.9%) | 24 (10.5%) | 42 (9.2%) |
| Intravenous Catheter | 9 (3.9%) | 10 (4.4%) | 19 (4.2%) |
| Central nervous system | 12 (5.3%) | 9 (3.9%) | 21 (4.6%) |
| Gut | 7 (3.1%) | 9 (3.9%) | 16 (3.5%) |
| Endocarditis | 4 (1.8%) | 1 (0.4%) | 5 (1.1%) |
| Other | 9 (3.9%) | 10 (4.4%) | 19 (4.2%) |
| Gynaecological | 0 (0.0%) | 1 (0.4%) | 1 (0.2%) |
| Intra thoracal | 2 (0.9%) | 0 (0.0%) | 2 (0.4%) |
| Musculoskeletal | 2 (0.9%) | 0 (0.0%) | 2 (0.4%) |
| Oro Naso pharyngal | 2 (0.9%) | 3 (1.3%) | 5 (1.1%) |
| Soft tissue | 1 (0.4%) | 1 (0.4%) | 2 (0.4%) |
| Unknown | 2 (0.9%) | 5 (2.2%) | 7 (1.5%) |
|  | | | |
|  | | | |
| **Third site of infection** | **n=44** | **n=38** | **n=82** |
| Pulmonary | 15 (34.1%) | 13 (34.2%) | 28 (34.1%) |
| Intra-abdominal | 5 (11.4%) | 4 (10.5%) | 9 (11.0%) |
| Blood | 6 (13.6%) | 8 (21.1%) | 14 (17.1%) |
| Skin | 14 (31.8%) | 6 (15.8%) | 20 (24.4%) |
| Urinary | 4 (9.1%) | 2 (5.3%) | 6 (7.3%) |
| Intravenous Catheter | 0 (0.0%) | 0 (0.0%) | 0 (0.0%) |
| Central nervous system | 0 (0.0%) | 0 (0.0%) | 0 (0.0%) |
| Gut | 0 (0.0%) | 2 (5.3%) | 2 (2.4%) |
| Endocarditis | 0 (0.0%) | 0 (0.0%) | 0 (0.0%) |
| Other | 0 (0.0%) | 3 (7.9%) | 3 (3.7%) |
| Musculoskeletal | 0 (0.0%) | 2 (5.3%) | 2 (2.4%) |
| Oro Naso pharyngal | 0 (0.0%) | 1 (2.6%) | 1 (1.2%) |
|  | | | |

**Supplementary Table 5: Infective Organisms Identified from the Primary Site of Infection**

|  | **Continuous Infusion (N = 1436)** | **Intermittent Infusion (N = 1464)** | **Total (N = 2900)** |
| --- | --- | --- | --- |
|  | | | |
| **Organism identified in primary site of infection** | **n=425** | **n=408** | **n=833** |
|  | | | |
|  | | | |
|  |  |  |  |
| **Gram +ve bacteria** | **129 (30.4%)** | **146 (35.8%)** | **275 (33.0%)** |
| Methicillin-Sensitive Staphylococcus aureus (MSSA) | 56 (13.2%) | 65 (15.9%) | 121 (14.5%) |
| Streptococcus pneumoniae (or Pneumococcus) | 21 (4.9%) | 26 (6.4%) | 47 (5.6%) |
| Beta-hemolytic streptococci (group A, B, C or G) | 15 (3.5%) | 10 (2.5%) | 25 (3.0%) |
| Coagulase negative staphylococcus | 12 (2.8%) | 12 (2.9%) | 24 (2.9%) |
| Enterococcus | 12 (2.8%) | 9 (2.2%) | 21 (2.5%) |
| Viridans Group Streptococci | 8 (1.9%) | 12 (2.9%) | 20 (2.4%) |
| Gram positive cocci not otherwise specified | 3 (0.7%) | 7 (1.7%) | 10 (1.2%) |
| Methicillin Resistant Staphylococcus aureus (MRSA) | 3 (0.7%) | 6 (1.5%) | 9 (1.1%) |
| Corynebacterium species | 1 (0.2%) | 2 (0.5%) | 3 (0.4%) |
| Bacillus species | 1 (0.2%) | 1 (0.2%) | 2 (0.2%) |
| Clostridioides difficile | 0 (0.0%) | 2 (0.5%) | 2 (0.2%) |
| Other Gram positive organisms | 2 (0.5%) | 0 (0.0%) | 2 (0.2%) |
|  |  |  |  |
| **Gram -ve bacteria** | **296 (69.6%)** | **255 (62.5%)** | **551 (66.1%)** |
| Escherichia species | 74 (17.4%) | 62 (15.2%) | 136 (16.3%) |
| Klebsiella species | 59 (13.9%) | 46 (11.3%) | 105 (12.6%) |
| Pseudomonas species | 57 (13.4%) | 44 (10.8%) | 101 (12.1%) |
| Haemophilus species | 26 (6.1%) | 21 (5.1%) | 47 (5.6%) |
| Enterobacter species | 18 (4.2%) | 22 (5.4%) | 40 (4.8%) |
| Serratia species | 12 (2.8%) | 15 (3.7%) | 27 (3.2%) |
| Citrobacter species | 11 (2.6%) | 10 (2.5%) | 21 (2.5%) |
| Proteus species | 8 (1.9%) | 7 (1.7%) | 15 (1.8%) |
| Coliform bacteria (genus not specified) | 7 (1.6%) | 6 (1.5%) | 13 (1.6%) |
| Acinetobacter species | 3 (0.7%) | 6 (1.5%) | 9 (1.1%) |
| Other Gram negative organisms | 4 (0.9%) | 5 (1.2%) | 9 (1.1%) |
| Morganella species | 4 (0.9%) | 4 (1.0%) | 8 (1.0%) |
| Gram negative rods not otherwise specified | 2 (0.5%) | 4 (1.0%) | 6 (0.7%) |
| Raoultella species | 3 (0.7%) | 3 (0.7%) | 6 (0.7%) |
| Stenotrophamonas species | 4 (0.9%) | 1 (0.2%) | 5 (0.6%) |
| Legionella species | 4 (0.9%) | 0 (0.0%) | 4 (0.5%) |
| Bacteroides species | 0 (0.0%) | 3 (0.7%) | 3 (0.4%) |
| Moraxella catarrhalis | 1 (0.2%) | 2 (0.5%) | 3 (0.4%) |
| Burkholderia species | 1 (0.2%) | 1 (0.2%) | 2 (0.2%) |
| Pantoea species | 1 (0.2%) | 1 (0.2%) | 2 (0.2%) |
| Hafnia alvei | 0 (0.0%) | 1 (0.2%) | 1 (0.1%) |
|  |  |  |  |
| **Other bacteria** | **11 (2.6%)** | **12 (2.9%)** | **23 (2.8%)** |
| Mixed anaerobes | 10 (2.4%) | 11 (2.7%) | 21 (2.5%) |
| Mycobacterium tuberculosis | 1 (0.2%) | 0 (0.0%) | 1 (0.1%) |
| Unknown | 0 (0.0%) | 1 (0.2%) | 1 (0.1%) |
|  | | | |

**Table Footn*ote***

*The percentages are based on those who had at least 1 organism identified in the primary site of infection.*

**Supplementary Table 6: Infective Organisms Identified from Second Site of Infection**

|  | **Continuous Infusion (N = 1436)** | **Intermittent Infusion (N = 1464)** | **Total (N = 2900)** |
| --- | --- | --- | --- |
|  | | | |
| **Organism identified in second site of infection** | **n=123** | **n=116** | **n=239** |
|  | | | |
|  | | | |
|  |  |  |  |
| **Gram +ve bacteria** | **49 (39.8%)** | **44 (37.9%)** | **93 (38.9%)** |
| Methicillin-Sensitive Staphylococcus aureus (MSSA) | 14 (11.4%) | 5 (4.3%) | 19 (7.9%) |
| Enterococcus | 7 (5.7%) | 10 (8.6%) | 17 (7.1%) |
| Coagulase negative staphylococcus | 9 (7.3%) | 6 (5.2%) | 15 (6.3%) |
| Streptococcus pneumoniae (or Pneumococcus) | 3 (2.4%) | 9 (7.8%) | 12 (5.0%) |
| Beta-hemolytic streptococci (group A, B, C or G) | 7 (5.7%) | 2 (1.7%) | 9 (3.8%) |
| Methicillin Resistant Staphylococcus aureus (MRSA) | 1 (0.8%) | 8 (6.9%) | 9 (3.8%) |
| Gram positive cocci not otherwise specified | 4 (3.3%) | 2 (1.7%) | 6 (2.5%) |
| Other Gram positive organisms | 3 (2.4%) | 1 (0.9%) | 4 (1.7%) |
| Cutibacterium species | 0 (0.0%) | 1 (0.9%) | 1 (0.4%) |
| Viridans Group Streptococci | 1 (0.8%) | 0 (0.0%) | 1 (0.4%) |
|  |  |  |  |
| **Gram -ve bacteria** | **72 (58.5%)** | **71 (61.2%)** | **143 (59.8%)** |
| Escherichia species | 24 (19.5%) | 19 (16.4%) | 43 (18.0%) |
| Klebsiella species | 16 (13.0%) | 17 (14.7%) | 33 (13.8%) |
| Enterobacter species | 8 (6.5%) | 6 (5.2%) | 14 (5.9%) |
| Pseudomonas species | 5 (4.1%) | 8 (6.9%) | 13 (5.4%) |
| Other Gram negative organisms | 3 (2.4%) | 4 (3.4%) | 7 (2.9%) |
| Haemophilus species | 4 (3.3%) | 2 (1.7%) | 6 (2.5%) |
| Citrobacter species | 3 (2.4%) | 2 (1.7%) | 5 (2.1%) |
| Gram negative rods not otherwise specified | 1 (0.8%) | 3 (2.6%) | 4 (1.7%) |
| Proteus species | 1 (0.8%) | 3 (2.6%) | 4 (1.7%) |
| Serratia species | 2 (1.6%) | 2 (1.7%) | 4 (1.7%) |
| Morganella species | 2 (1.6%) | 1 (0.9%) | 3 (1.3%) |
| Acinetobacter species | 2 (1.6%) | 0 (0.0%) | 2 (0.8%) |
| Bacteroides species | 1 (0.8%) | 1 (0.9%) | 2 (0.8%) |
| Coliform bacteria (genus not specified) | 1 (0.8%) | 0 (0.0%) | 1 (0.4%) |
| Fusobacterium species | 0 (0.0%) | 1 (0.9%) | 1 (0.4%) |
| Moraxella catarrhalis | 0 (0.0%) | 1 (0.9%) | 1 (0.4%) |
| Stenotrophamonas species | 0 (0.0%) | 1 (0.9%) | 1 (0.4%) |
|  |  |  |  |
| **Other bacteria** | **2 (1.6%)** | **1 (0.9%)** | **3 (1.3%)** |
| Mixed anaerobes | 2 (1.6%) | 1 (0.9%) | 3 (1.3%) |
|  | | | |

**Table Footn*ote****:*

*The percentages are based on those who had at least 1 organism identified in the second site of infection.*

**Supplementary Table 7: Adverse Events**

|  | **Continuous Infusion (N=1421)** | **Intermittent Infusion (N=1440)** | **Total (N=2861)** | **p-value** |
| --- | --- | --- | --- | --- |
|  | | | | |
| **Adverse Events** | 6^#^ 6 (0.4%) | 5^#^ 5 (0.3%) | 11^#^ 11 (0.4%) | 0.7726 |
|  |  |  |  |  |
| **Adverse Events Categories** |  |  |  |  |
| Elevated liver enzyme/s | 1 (0.03%) | 0 | 1 (0.01%) |  |
| Fever | 2 (0.06%) | 0 | 2 (0.03%) |  |
| Hypertension/hypotension | 0 | 1 (0.03%) | 1 (0.01%) |  |
| Post-operative bleeding | 0 | 1 (0.03%) | 1 (0.01%) |  |
| Rash | 3 (0.09%) | 1 (0.03%) | 4 (0.06%) |  |
| Rash, tachycardia, hypotension | 0 0 | 1 (0.03%) | 1 (0.01%) |  |
| Redness/pain at IV insertion site | 0 0 | 1 (0.03%) | 1 (0.01%) |  |
|  | | | | |
|  | | | | |
| **Serious Adverse Events** | 0 | 0 | 0 |  |
|  |  |  |  |  |
| **Serious Criteria** |  |  |  |  |
| Resulted in death | 0 | 0 | 0 |  |
| Life threatening | 0 | 0 | 0 |  |
| Requires prolonged hospitalisation | 0 | 0 | 0 |  |
| Results in persistent or severe disability/incapacity | 0 | 0 | 0 |  |
| Results in congenital anomaly/birth defect | 0 | 0 | 0 |  |
| Medically significant to qualify as a serious event | 0 | 0 | 0 |  |
|  | | | | |
|  | | | | |
| **Relationship to study treatment** |  |  |  |  |
| Possibly related | 4^#^ 4 (0.3%) | 4^#^ 4 (0.3%) | 8^#^ 8 (0.3%) |  |
| Probably related | 2^#^ 2 (0.1%) | 1^#^ 1 (0.1%) | 3^#^ 3 (0.1%) |  |
| Definitely related | 0 | 0 | 0 |  |
|  | | | | |

**Table Footn*ote***

*AEs are summarised as the total number of events reported^#^ followed by the number and percentage of patients having at least one event.
The denominators for percentages are all randomised patients.
p-values are by Fisher exact test.*

**Supplementary Table 8: Other antibiotics administered in the 24 hours before randomisation and up to day 16**

| **Antibiotics** | **Continuous Infusion (N = 1436)** | **Intermittent Infusion (N = 1464)** | **Total (N = 2900)** |
| --- | --- | --- | --- |
|  | | | |
| Any other antibiotics | 1147 (79.9%) | 1184 (80.9%) | 2331 (80.4%) |
|  | | | |
|  | | | |
| Amoxicillin/clavulanic acid (Augmentin) | 430 (29.9%) | 434 (29.6%) | 864 (29.8%) |
| Gentamicin | 297 (20.7%) | 340 (23.2%) | 637 (22.0%) |
| Metronidazole | 253 (17.6%) | 250 (17.1%) | 503 (17.3%) |
| Vancomycin | 259 (18.0%) | 229 (15.6%) | 488 (16.8%) |
| Clarithromycin | 150 (10.4%) | 183 (12.5%) | 333 (11.5%) |
| Amikacin | 151 (10.5%) | 168 (11.5%) | 319 (11.0%) |
| Ciprofloxacin | 105 (7.3%) | 94 (6.4%) | 199 (6.9%) |
| Trimethoprim-Sulfamethoxazole | 85 (5.9%) | 102 (7.0%) | 187 (6.4%) |
| Amoxicillin/Ampicillin | 86 (6.0%) | 96 (6.6%) | 182 (6.3%) |
| Ceftriaxone | 84 (5.8%) | 90 (6.1%) | 174 (6.0%) |
| Teicoplanin | 82 (5.7%) | 92 (6.3%) | 174 (6.0%) |
| Flucloxacillin | 88 (6.1%) | 85 (5.8%) | 173 (6.0%) |
| Linezolid | 89 (6.2%) | 66 (4.5%) | 155 (5.3%) |
| Clindamycin | 67 (4.7%) | 76 (5.2%) | 143 (4.9%) |
| Erythromycin | 55 (3.8%) | 83 (5.7%) | 138 (4.8%) |
| Doxycycline | 42 (2.9%) | 53 (3.6%) | 95 (3.3%) |
| Cefuroxime | 46 (3.2%) | 44 (3.0%) | 90 (3.1%) |
| Levofloxacin | 28 (1.9%) | 33 (2.3%) | 61 (2.1%) |
| Penicillin (Penicillin G or Benzylpenicillin) | 25 (1.7%) | 33 (2.3%) | 58 (2.0%) |
| Ceftazidime | 19 (1.3%) | 28 (1.9%) | 47 (1.6%) |
| Azithromycin | 12 (0.8%) | 14 (1.0%) | 26 (0.9%) |
| Rifampicin | 13 (0.9%) | 13 (0.9%) | 26 (0.9%) |
| Aztreonam | 9 (0.6%) | 13 (0.9%) | 22 (0.8%) |
| Temocillin | 12 (0.8%) | 10 (0.7%) | 22 (0.8%) |
| Rifaximin | 6 (0.4%) | 12 (0.8%) | 18 (0.6%) |
| Phenoxymethylpenicillin | 7 (0.5%) | 9 (0.6%) | 16 (0.6%) |
| Moxifloxacin | 5 (0.3%) | 10 (0.7%) | 15 (0.5%) |
| Cefotaxime | 3 (0.2%) | 8 (0.5%) | 11 (0.4%) |
| Tigecycline | 4 (0.3%) | 7 (0.5%) | 11 (0.4%) |
| Ertapenem | 5 (0.3%) | 5 (0.3%) | 10 (0.3%) |
| Other | 8 (0.6%) | 2 (0.1%) | 10 (0.3%) |
| Gatifloxacin | 2 (0.1%) | 0 (0.0%) | 2 (0.1%) |
| Neomycin | 1 (0.1%) | 1 (0.1%) | 2 (0.1%) |
| Ticarcillin/clavulanic acid (Timentin) | 2 (0.1%) | 0 (0.0%) | 2 (0.1%) |
| Cefaclor | 1 (0.1%) | 0 (0.0%) | 1 (0.0%) |
| Cefoxitin | 1 (0.1%) | 0 (0.0%) | 1 (0.0%) |
| Fosfomycin | 0 (0.0%) | 1 (0.1%) | 1 (0.0%) |
| Lymecycline | 1 (0.1%) | 0 (0.0%) | 1 (0.0%) |
| Chloramphenicol | 4 (0.3%) | 2 (0.1%) | 6 (0.2%) |
| Fidaxomicin | 2 (0.1%) | 3 (0.2%) | 5 (0.2%) |
| Pyrazinamide | 0 (0.0%) | 5 (0.3%) | 5 (0.2%) |
| Daptomycin | 1 (0.1%) | 3 (0.2%) | 4 (0.1%) |
| Nitrofurantoin | 1 (0.1%) | 3 (0.2%) | 4 (0.1%) |
| Colistin | 1 (0.1%) | 2 (0.1%) | 3 (0.1%) |
| Ethambutol | 0 (0.0%) | 2 (0.1%) | 2 (0.1%) |
| Cefazolin | 1 (0.1%) | 0 (0.0%) | 1 (0.0%) |
| Spiramycin | 0 (0.0%) | 1 (0.1%) | 1 (0.0%) |
| Tobramycin | 0 (0.0%) | 1 (0.1%) | 1 (0.0%) |
| Isoniazid | 2 (0.1%) | 8 (0.5%) | 10 (0.3%) |
|  | | | |

**Supplementary Table 9: Place and cause of death**

| **Characteristics** | **Continuous Infusion (N = 1421)** | **Intermittent Infusion (N = 1440)** | **Total (N = 2861)** | **p-value** |
| --- | --- | --- | --- | --- |
|  | | | | |
| **Place of death** |  |  |  | 0.8054 |
| ICU (including intensivist supervised HDU) | 249/373 (66.8%) | 289/415 (69.6%) | 538/788 (68.3%) |  |
| Ward (including HDU if NOT supervised by intensivist) | 99/373 (26.5%) | 100/415 (24.1%) | 199/788 (25.3%) |  |
| Other | 16/373 (4.3%) | 15/415 (3.6%) | 31/788 (3.9%) |  |
| Home | 9/373 (2.4%) | 11/415 (2.7%) | 20/788 (2.5%) |  |
|  | | | | |
|  | | | | |
| **Proximate cause of death** | 371 | 408 | 779 |  |
| Hypoxic respiratory failure | 85/371 (22.9%) | 88/408 (21.6%) | 173/779 (22.2%) |  |
| Distributive (Septic) shock | 42/371 (11.3%) | 62/408 (15.2%) | 104/779 (13.4%) |  |
| Neurological no TBI without brain death | 31/371 (8.4%) | 26/408 (6.4%) | 57/779 (7.3%) |  |
| Neurological TBI without brain death | 18/371 (4.9%) | 14/408 (3.4%) | 32/779 (4.1%) |  |
| Neurological no TBI with brain death | 13/371 (3.5%) | 15/408 (3.7%) | 28/779 (3.6%) |  |
| Neurological TBI with brain death | 6/371 (1.6%) | 9/408 (2.2%) | 15/779 (1.9%) |  |
| Metabolic | 6/371 (1.6%) | 9/408 (2.2%) | 15/779 (1.9%) |  |
| Cardiogenic shock | 6/371 (1.6%) | 8/408 (2.0%) | 14/779 (1.8%) |  |
| Arrhythmia | 9/371 (2.4%) | 2/408 (0.5%) | 11/779 (1.4%) |  |
| Hypovolaemic shock | 5/371 (1.3%) | 2/408 (0.5%) | 7/779 (0.9%) |  |
| Other | 150/371 (40.4%) | 173/408 (42.4%) | 323/779 (41.5%) |  |
|  | | | | |
|  | | | | |
| **Underlying cause of death** | 371 | 407 | 778 |  |
| ***Neurological Cause*** |  |  |  |  |
| Hypoxic brain injury | 25/371 (6.7%) | 21/407 (5.2%) | 46/778 (5.9%) |  |
| Other neurological condition | 17/371 (4.6%) | 19/407 (4.7%) | 36/778 (4.6%) |  |
| Haemorrhagic stroke | 8/371 (2.2%) | 19/407 (4.7%) | 27/778 (3.5%) |  |
| Aneurysmal SAH | 12/371 (3.2%) | 15/407 (3.7%) | 27/778 (3.5%) |  |
| Ischaemic stroke | 12/371 (3.2%) | 13/407 (3.2%) | 25/778 (3.2%) |  |
| Traumatic brain injury (unsurvivable primary injury) | 10/371 (2.7%) | 10/407 (2.5%) | 20/778 (2.6%) |  |
| Metabolic encephalopathy | 3/371 (0.8%) | 3/407 (0.7%) | 6/778 (0.8%) |  |
| Traumatic brain injury (refractory intracranial hypertension) | 1/371 (0.3%) | 2/407 (0.5%) | 3/778 (0.4%) |  |
| Meningoencephalitis | 1/371 (0.3%) | 2/407 (0.5%) | 3/778 (0.4%) |  |
| Status epilepticus | 1/371 (0.3%) | 2/407 (0.5%) | 3/778 (0.4%) |  |
| Cerebral abscess | 0/371 (0.0%) | 0/407 (0.0%) | 0/778 (0.0%) |  |
|  |  |  |  |  |
| ***Cardiovascular Cause*** |  |  |  |  |
| Sepsis with multi-organ failure | 70/371 (18.9%) | 83/407 (20.4%) | 153/778 (19.7%) |  |
| Other cardiovascular condition | 44/371 (11.9%) | 58/407 (14.3%) | 102/778 (13.1%) |  |
| Hepatic Failure | 21/371 (5.7%) | 24/407 (5.9%) | 45/778 (5.8%) |  |
| AMI | 11/371 (3.0%) | 15/407 (3.7%) | 26/778 (3.3%) |  |
| Haemorrhage not due to trauma | 11/371 (3.0%) | 8/407 (2.0%) | 19/778 (2.4%) |  |
| Pancreatitis | 8/371 (2.2%) | 3/407 (0.7%) | 11/778 (1.4%) |  |
| Aortic valvular disease | 4/371 (1.1%) | 2/407 (0.5%) | 6/778 (0.8%) |  |
| Massive pulmonary thromboembolism | 4/371 (1.1%) | 1/407 (0.2%) | 5/778 (0.6%) |  |
| Ruptured or leaking AAA | 0/371 (0.0%) | 2/407 (0.5%) | 2/778 (0.3%) |  |
| Haemorrhage due to trauma | 0/371 (0.0%) | 1/407 (0.2%) | 1/778 (0.1%) |  |
| Mitral valve disease | 0/371 (0.0%) | 1/407 (0.2%) | 1/778 (0.1%) |  |
| Myocarditis | 0/371 (0.0%) | 0/407 (0.0%) | 0/778 (0.0%) |  |
| Ruptured or leaking thoracic AA | 0/371 (0.0%) | 0/407 (0.0%) | 0/778 (0.0%) |  |
| Pericardial tamponade | 0/371 (0.0%) | 0/407 (0.0%) | 0/778 (0.0%) |  |
| Anaphylaxis | 0/371 (0.0%) | 0/407 (0.0%) | 0/778 (0.0%) |  |
|  |  |  |  |  |
| ***Respiratory Cause*** |  |  |  |  |
| Pneumonia | 99/371 (26.7%) | 93/407 (22.9%) | 192/778 (24.7%) |  |
| Cancer | 47/371 (12.7%) | 56/407 (13.8%) | 103/778 (13.2%) |  |
| Other respiratory condition | 32/371 (8.6%) | 37/407 (9.1%) | 69/778 (8.9%) |  |
| COPD | 20/371 (5.4%) | 34/407 (8.4%) | 54/778 (6.9%) |  |
| ARDS- pulmonary | 17/371 (4.6%) | 10/407 (2.5%) | 27/778 (3.5%) |  |
| Aspiration pneumonitis | 12/371 (3.2%) | 9/407 (2.2%) | 21/778 (2.7%) |  |
| Pulmonary fibrosis | 7/371 (1.9%) | 6/407 (1.5%) | 13/778 (1.7%) |  |
| Asthma | 2/371 (0.5%) | 3/407 (0.7%) | 5/778 (0.6%) |  |
| Pulmonary haemorrhage | 0/371 (0.0%) | 1/407 (0.2%) | 1/778 (0.1%) |  |
| ARDS- non-pulmonary | 0/371 (0.0%) | 0/407 (0.0%) | 0/778 (0.0%) |  |
|  |  |  |  |  |
| ***Metabolic Cause*** |  |  |  |  |
| Diabetes | 23/371 (6.2%) | 22/407 (5.4%) | 45/778 (5.8%) |  |
| Renal Failure | 22/371 (5.9%) | 22/407 (5.4%) | 44/778 (5.7%) |  |
| Vasculitis | 1/371 (0.3%) | 2/407 (0.5%) | 3/778 (0.4%) |  |
| Hepatitis | 2/371 (0.5%) | 0/407 (0.0%) | 2/778 (0.3%) |  |
| Drug induced | 2/371 (0.5%) | 0/407 (0.0%) | 2/778 (0.3%) |  |
| Drug Overdose | 0/371 (0.0%) | 1/407 (0.2%) | 1/778 (0.1%) |  |
| Anorexia/Cachexia | 0/371 (0.0%) | 1/407 (0.2%) | 1/778 (0.1%) |  |
| Hypoadrenalism | 0/371 (0.0%) | 0/407 (0.0%) | 0/778 (0.0%) |  |
|  |  |  |  |  |
| ***Other Cause*** |  |  |  |  |
| Other cause not listed | 102/371 (27.5%) | 119/407 (29.2%) | 221/778 (28.4%) |  |

**Table Footn*ote***

*These causes are from a predetermined list for trial purposes, not the from the Medical Certificate of Cause of Death*

*Fisher exact test is used to test the differences in distribution between the two treatment arms.*

**Supplementary Table 10: Reason prescription stopped**

| **Reasons** | **Continuous Infusion (N = 1436)** | **Intermittent Infusion (N = 1464)** | **Total (N = 2900)** |
| --- | --- | --- | --- |
|  | | | |
| **Reason prescription stopped** | n=1414 | n=1441 | n=2855 |
| Treatment course of beta-lactam antibiotic completed | 386 (27.3%) | 658 (45.7%) | 1044 (36.6%) |
| Change in antimicrobial therapy | 945 (66.8%) | 619 (43.0%) | 1564 (54.8%) |
| Adverse event or contraindication to beta-lactam antibiotic | 4 (0.3%) | 5 (0.3%) | 9 (0.3%) |
| Patient treatment focus changed to palliation | 25 (1.8%) | 70 (4.9%) | 95 (3.3%) |
| Discharge from ICU | 54 (3.8%) | 89 (6.2%) | 143 (5.0%) |
|  | | | |

**Supplementary Figure**

**
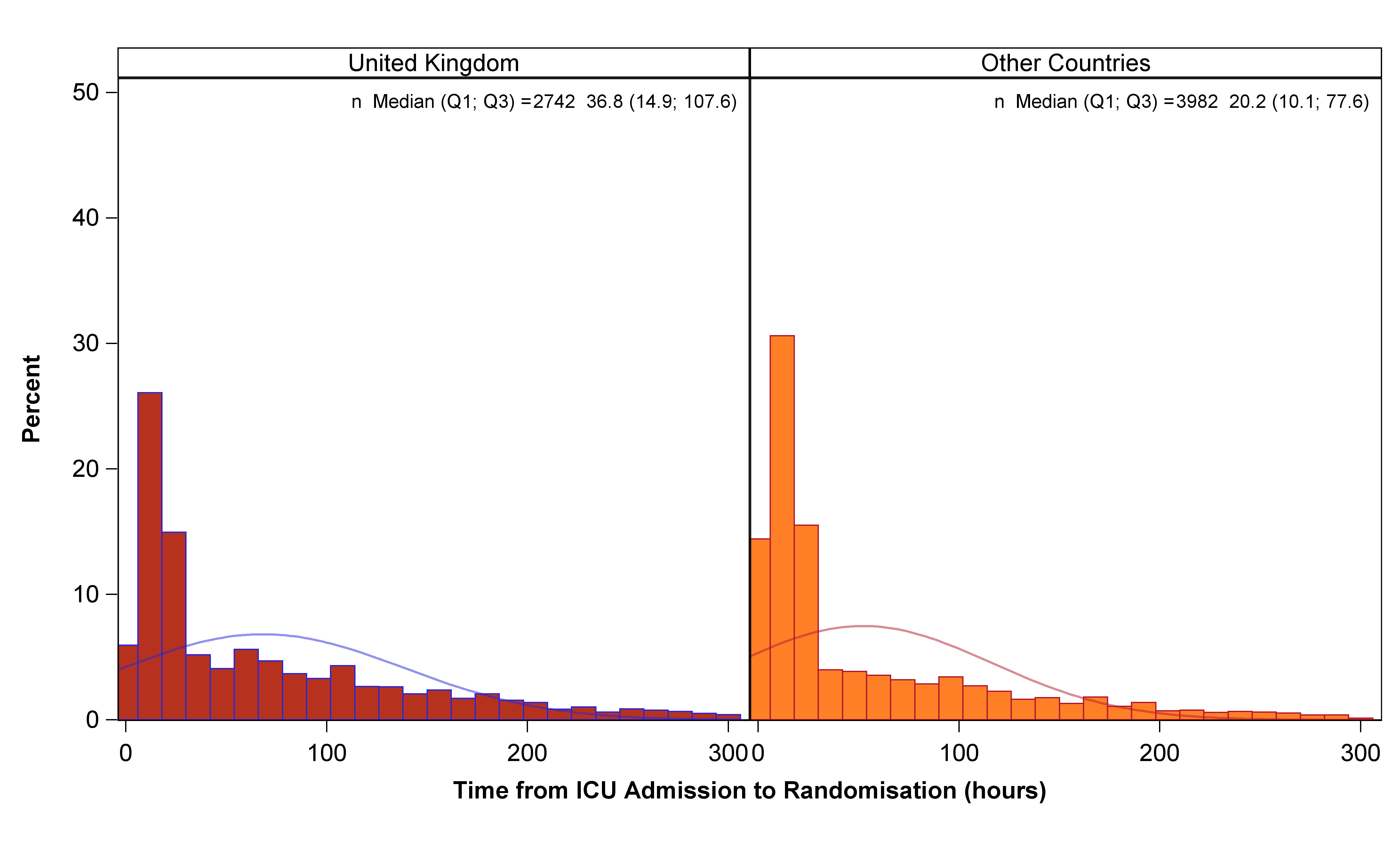
**

**Figure Footnote**

Curves traversing the histograms are normal density estimates, to assist in visualising degree of skewness.

RESERVED
